# The role of childhood social position in adult type 2 diabetes: evidence from the English Longitudinal Study of Ageing

**DOI:** 10.1186/1471-2458-14-505

**Published:** 2014-05-26

**Authors:** Jitka Pikhartova, David Blane, Gopalakrishnan Netuveli

**Affiliations:** 1 School of Health Sciences and Social Care, Brunel University, Uxbridge UB8 3PH, Middlesex, UK; 2 Department of Primary Care and Public Health, Imperial College London, London W6 8RP, UK; 3 Institute of Health and Human Development, University of East London, London E15 4LZ, UK

**Keywords:** ELSA, Diabetes, Longitudinal, Glycated haemoglobin, Inflammation

## Abstract

**Background:**

Socioeconomic circumstances in childhood and early adulthood may influence the later onset of chronic disease, although such research is limited for type 2 diabetes and its risk factors at the different stages of life. The main aim of the present study is to examine the role of childhood social position and later inflammatory markers and health behaviours in developing type 2 diabetes at older ages using a pathway analytic approach.

**Methods:**

Data on childhood and adult life circumstances of 2,994 men and 4,021 women from English Longitudinal Study of Ageing (ELSA) were used to evaluate their association with diabetes at age 50 years and more. The cases of diabetes were based on having increased blood levels of glycated haemoglobin and/or self-reported medication for diabetes and/or being diagnosed with type 2 diabetes. Father’s job when ELSA participants were aged 14 years was used as the measure of childhood social position. Current social characteristics, health behaviours and inflammatory biomarkers were used as potential mediators in the statistical analysis to assess direct and indirect effects of childhood circumstances on diabetes in later life.

**Results:**

12.6 per cent of participants were classified as having diabetes. A disadvantaged social position in childhood, as measured by father’s manual occupation, was associated at conventional levels of statistical significance with an increased risk of type 2 diabetes in adulthood, both directly and indirectly through inflammation, adulthood social position and a risk score constructed from adult health behaviours including tobacco smoking and limited physical activity. The direct effect of childhood social position was reduced by mediation analysis (standardised coefficient decreased from 0.089 to 0.043) but remained statistically significant (p = 0.035). All three indirect pathways made a statistically significantly contribution to the overall effect of childhood social position on adulthood type 2 diabetes.

**Conclusions:**

Childhood social position influences adult diabetes directly and indirectly through inflammatory markers, adulthood social position and adult health behaviours.

## Background

Early life socio-demographic characteristics and their influence on the onset of adult chronic disease have become recently the focus of more intensive research [1], with the findings showing that a disadvantaged social position in childhood is associated with higher morbidity and mortality later in life [2,3].

Diabetes mellitus is a serious disease affecting a significant proportion of the population [1]; and its incidence and prevalence are increasing (in Britain, for example, its prevalence increased between 1995 and 2006 from 2.8% to 4.5%) [4]. A wide range of risk factors for diabetes have been proposed, including age, ethnicity, family history, intra-uterine growth, childhood health and obesity and, later in life, BMI or waist circumference and physical activity, although the results sometimes have been inconsistent [5-11]. The role of tobacco smoking is unclear [12], with some studies finding an indirect effect of smoking on the development of glucose intolerance [13].

The association between adulthood socioeconomic position and diabetes morbidity and mortality is well-known [14], although it may differ by gender, and some studies did not find such association at older ages [15]. The role of adverse childhood social environment in development of increased insulin resistance or diabetes is less clear. Direct association between social position in childhood and increased insulin resistance has been found only very rarely in the previous research [16] and some studies have questioned such association mainly because the association between childhood social conditions and diabetes was usually explained by socioeconomic position in adulthood [10,17,18]. Few studies proposed pathways through which adverse childhood environment influenced later onset of type 2 diabetes indirectly, such as through stress originating from poorer living conditions in childhood, earlier health problems, financial security or family stability, or through unhealthy behaviours and overweight [8,19-23]. Inflammation has also been proposed as possible link between low social status and type 2 diabetes [24]. Inflammatory markers were found elevated in those with risky health behaviours which in turn are closely associated with lower socioeconomic position and stressful life and also with type 2 diabetes [24]. Stress can stimulate HPA axis which can stimulate higher production of cortisol and consequently influence levels of inflammatory markers, production of insulin or adipose tissues gain [24,25]. Several studies showed the association between inflammatory markers and type 2 diabetes [26,27].

The concepts of life-course epidemiology can be used to investigate the influence of factors in childhood and adulthood on the later onset of diabetes and other diseases. It can be used to identify particularly the sensitive periods where socioeconomic factors play a role in the development of diabetes. It can be useful also for evaluating the magnitude of any direct association between childhood circumstances and the later onset of diabetes; as well as to describe a series of life stages and help to assess whether the role of socioeconomic exposures accumulates over time and across the life-cycle. This life course methodology has been used by many researchers to evaluate the effect of adversity in childhood and other life stages on the risk of later disease onset [28,29], but it has been used little in diabetes research.

In the present paper we use data from the English Longitudinal Study of Ageing (ELSA). The main aim is to examine the role of childhood and adulthood socioeconomic position, inflammatory markers and health behaviours in developing type 2 diabetes at older ages using a life course analytical approach in a sample of men and women aged 50 years and more. The life course approach has been used little in diabetes research and has not been used previously to analyse diabetes outcomes in ELSA. We use multiple imputations to maximise the effective sample size and path analysis to assess direct and indirect effects.

## Methods

### Study population and study sample

Data for this analysis were derived from the English Longitudinal Study of Ageing (ELSA). The ELSA sample consists of people aged 50 years and more who were selected from participants in the Health Surveys for England (HSE) in 1998, 1999 and 2001, constructed to represent the English population over 50 years of age living in private addresses. Those who agreed to participate were invited to wave 1 of ELSA in 2002–03, with subsequent follow-up every two years. Wave 1 data were collected by interviews alone, but Waves 2 and 4 included physical examinations and blood samples collection. Ethical approval for all the ELSA waves was granted by the National Research and Ethics Committee. All participants gave informed consent. More information on ELSA can be found at http://www.ifs.org.uk/elsa/documentation.php. This analysis includes data from all four waves (including nurse’s part of wave 4). Data were accessed through the Economic and Social Data Service.

The present study sample consists of the 7,015 men and women, from the original 11,392 ELSA members, who were screened at Waves 1 and 4 and had valid outcome data (described in the next section). Loss to follow-up in ELSA is associated with male gender, morbidity, older age and a disadvantaged social position [30]. Nurses invited some three-quarters of Wave 4 participants to give a blood sample, excluding those who refused informed consent or who had a history of fits or convulsion, bleeding or clotting disorders or were prescribed anticoagulants. Glycated haemoglobin (HbA1c) samples were finally available for 4,245 individuals of 7,015 individuals in the analytical sample. All participants from English Longitudinal Study of Ageing signed full informed consent to participate in the study.

### Construction of diabetes diagnosis variable

The outcome variable was based on combination of the following four conditions. Individuals were classified as having diabetes if the level of glycated haemoglobin (Hb1Ac) exceeded 6.5% (>48 mmol/mol, following International Diabetes Federation recommendations) when measured in wave 4 and/or if they answered positively at least one of the three following questions: “Has a doctor ever told you that you have diabetes or high blood sugar?”, “Do you currently inject insulin for diabetes?”, and “Are you currently taking any tablets, pills or other medicines that you swallow for diabetes?”. Fasting plasma glucose was not used as there was insufficient response to the fasting glucose blood test compared to HbA1c blood test, which did not require fasting. This self-reported information was derived from all four waves of ELSA.

The ascertainment of diabetes cases in ELSA in this analysis is as complete as possible with the available data. Using only the self-reported doctor diagnosis, one case in three would be missed.

### Explanatory variables characterizing childhood and adulthood

A range of variables characterizing childhood and adulthood (including current life) was used in the analysis. The core part of the ELSA dataset supplied the father’s occupation when the participant was aged 14 years, used as a dichotomous measure of the manual employment of the father. Other explanatory variables used to obtain information about adulthood and current life and health status included current risk factor score and recent social position.

The current risk factor score was constructed from: waist circumference measured during nurse examination at wave 4; information about level of physical activity collected at wave 4; and tobacco smoking assessed across all four waves. Guided by the literature that fat tissue behaves as an independent organ, producing hormones that contribute to systemic inflammation and the development of insulin-resistance, and that central obesity is sufficient instrument to measure the risk of being overweight [31,32], only waist measurement was used in our analysis. The current risk factor score was constructed as gender and age specific.

C-reactive protein and blood fibrinogen from wave 4 were grouped into deciles, coded 0–9. The inflammatory score was constructed combining C-reactive protein and blood fibrinogen decile scores.

### Statistical methods

Descriptive characteristics were obtained for an analytical sample including missing data. For the level of missingness in these data, 20 imputations are considered sufficient, so 20 imputed datasets were produced using Bayesian imputation methods in MPlus version 6.12. Afterwards a sensitivity analysis was performed on both the original and imputed datasets, to check that the imputation had not introduced bias. Logistic regression was used to assess bivariate associations between diabetes and the socioeconomic and health variables from childhood and adulthood. Age and sex- adjusted estimates of odds ratios (OR) were calculated together with 95% confidence intervals (CI). Sex interaction was tested in all steps of the analysis, and as there was not any statistically significant differences between the results for men and women, our results are being presented as sex- adjusted rather than stratified by sex. First, the association between childhood socioeconomic position and type 2 diabetes at older ages was assessed. Then, the association between the adult factors (inflammatory score, risk factor score, and socioeconomic position) and both childhood socioeconomic position and type 2 diabetes was assessed. All descriptive and regression analysis was done in STATA 11/12. The possible mediating role of inflammatory markers, current risk factors and current social position was evaluated in pathway analysis in MPlus version 6.12. Pathway analysis allowed assessing the direct and indirect relationship between childhood socioeconomic position and type 2 diabetes using several mediators simultaneously.

## Results

Data were available for 7,015 study participants after applying all eliminating processes. In comparison with the overall sample, the subgroup used in the present analyses was slightly older (68.9 years compared to 65.2 in the Wave 4 core sample); had slightly less males (42.7 per cent in our sample compared to 44.9%); the distribution of current social position was similar to the main dataset, with slightly higher proportion of males in the most disadvantaged social class (21.1 per cent compared to 19.3%) and a lower proportion of females in the same class (17.7 per cent compared to 20.9%). The prevalence of type 2 diabetes in the Wave 4 core sample, and in our sub-sample, and the prevalence in England in 2009 did not differ substantially (males in England respectively 14.2 per cent, 14.8% and 14.1%; in females 10.6%, 11.0% and 11.1%).

There were 385 respondents (5.5 per cent of the whole sample, 9% of those with valid measurements) with HbA1C levels greater than 6.5 per cent. 499 respondents reported taking tablets and 146 reported taking insulin in injections as their diabetes medication. 753 respondents reported they had been told in the past that they have diabetes or high blood sugar. Using the diabetes definition described in the Methods section, there were 886 (12.6%) respondents classified as having a type 2 diabetes in the sample. Table 1 shows the characteristics of the sample used in this data analysis before data imputation. There were 14.8% men and 11.0% women reporting diabetes. Rates of diabetes varied between 7.7% among those younger than 60 years and 15.6% among those older than 70. According to current social position, the highest rate of diabetes was among those in unskilled jobs (18.7%, 21.1% in men and 17.7% in women). There was large difference in the rate of diabetes by waist circumference with 17.1% of those with large waist circumference having diabetes (19.3% of men and 15.7% of women). Eighteen per cent of respondents with higher levels of CRP and 13% of those with higher fibrinogen levels were classified as having diabetes. In every population sub-group of population according to current or childhood factors there was a higher rate of diabetes among men.

**Table 1:** Distribution of social and demographic characteristics in the analytical sample, English Longitudinal Study of Ageing 2002-2008

		**Total**		**Males**		**Females**	
		**N (%)**	**Diabetes%**	**N**	**Diabetes%**	**N**	**Diabetes%**
		**7,015**	**12.6**	**2,994 (42.7%)**	**14.8**	**4,021 (57.3%)**	**11.0**
Age	<60	1,603 (22.9)	7.7	616	10.7	987	5.9
	61-70	2,526 (36.0)	12.3	1,153	14.2	1,373	10.7
	71+	2,886 (41.1)	15.6	1,225	17.4	1,661	14.3
Childhood social position	Non-manual father’s occupation	2,223 (31.7)	9.9	905	13.2	1,318	7.6
	Manual father’s occupation	4,350 (62.1)	13.9	1,914	15.8	2,436	12.5
	*Not available*	442 (6.3)		175		267	
Current social position	Professional	409 (5.8)	8.1	315	8.9	94	5.3
	Managerial technical	2,126 (30.3)	10.4	1,040	12.8	1,086	8.0
	Skilled	2,935 (41.8)	13.0	1,145	16.8	1,790	10.6
	Semi-skilled manual	999 (14.2)	14.9	341	17.6	658	13.5
	Unskilled	427 (6.1)	18.7	128	21.1	299	17.7
	*Not available*	119 (1.7)		25		94	
High waist circumference (cm)	>102 cm (M)	3,371 (48.1)	17.1	1.305	19.3	2,066	15.7
	>88 cm (F)						
	<102 cm (M)	2,422 (34.6)	6.7	1,217	9.5	1,205	3.9
	<88 cm (F)						
	*Not available*	1,222 (17.4)		472		750	
Physical activity	Yes	1,699 (24.2)	6.8	820	7.9	879	5.7
	No	5,313 (75.7)	14.5	2,173	17.4	3,140	12.5
	*Not available*	3 (0.04)		1		2	
Smoking	Yes	4,336 (61.8)	13.8	2,123	16.1	2,204	11.4
	No	2,671 (38.1)	10.8	859	11.5	1,812	10.5
	*Not available*	8 (0.1)		3		5	
C-reactive protein	Yes	165 (2.4)	18.2	63	22.2	102	15.7
	(>15 mg/l)						
	No	4,136 (59)	11.8	1,795	13.4	2,341	10.6
	*Not available*	2,714 (38.7)		1,136		1.578	
Fibrinogen	Yes (>3.00 g/l)	3,040 (43.3)	12.9	1,224	15.0	1,816	11.4
	No	1,143 (16.3)	9.8	582	10.0	561	9.6
	*Not available*	2,832 (40.4)		1,188		1,644	

Table 2 shows bivariate associations between variables characterizing childhood and adulthood and type 2 diabetes in later life. The direct association between disadvantaged social position in childhood and having type 2 diabetes in later life has the same strength as the association between current social position and type 2 diabetes diagnosis. Those who came from the most disadvantaged social background, or live in it currently, are respectively 1.45 times and 1.43 times more likely to have diabetes or high blood sugar in later life. The strongest association between the variables of interest is between disadvantaged social position in current life and the probability that inflammatory markers would be significantly raised (2.13 times more likely than those who have an advantaged social position).

**Table 2:** Bivariate associations between variables used in pathway model (Age-sex adjusted OR, 95% CI and level of significance)

**Exposure**	**Outcome**	**OR (95% CI)**	**p-value**
Childhood social position at age 14	Type 2 diabetes	1.45 (1.23-1.71)	<0.001
	Current social position^1^	2.84 (2.44-3.31)	<0.001
	Current risk factor score	1.47 (1.27-1.69)	<0.001
	Inflammatory markers score	1.63 (1.43-1.86)	<0.001
Current social position^1^	Type 2 diabetes	1.43 (1.22-1.69)	<0.001
Current risk factors score	Type 2 diabetes	1.33 (1.28-1.38)	<0.001
Inflammatory markers score	Type 2 diabetes	1.62 (1.34-1.96)	<0.001

^1^dichotomised as manual (semi-skilled manual and unskilled) and non-manual (professional, managerial technical and skilled).

Table 3 shows results of three pathway models. The first is simple direct model without any mediators, while the second model includes the inflammatory markers score as potential mediator between childhood social position and type 2 diabetes, and the third model uses all three possible pathways. The first model, of direct association, shows a positive relationship between childhood socioeconomic position and being diagnosed with type 2 diabetes in later life. The standardised coefficient related to the direct path from childhood social position to diabetes reduces in each step of the analysis and changes from 0.089 in first model to 0.043 in the third model, although always it remains statistically significant. The results show that current risk factor score mediates the main relationship between childhood social position and later onset of type 2 diabetes. The data suggest that childhood social position influences diabetes in adulthood both directly and indirectly through inflammatory markers, adulthood social position and adult behaviours grouped in a current risk score.

**Table 3:** Model estimates and model fit statistics for direct and indirect associations between childhood SEP and diabetes mellitus in older age

	**Direct Association**	**Indirect association – mediation through inflammation**			**Indirect Association – Mediation through Current SEP, Current Risk Factors and Inflammation**						
	**Type 2 diabetes on:**	**Inflammation on:**	**Type 2 diabetes on:**		**Current SEP on:**	**Current risk factors on:**	**Inflammation on:**	**Type 2 diabetes on:**			
	**Childhood SEP**	**Childhood SEP**	**Inflammation**	**Childhood SEP**	**Childhood SEP**	**Childhood SEP**	**Childhood SEP**	**Inflammation**	**Childhood SEP**	**Current SEP**	**Current risk factors**
Coefficient	0.191	0.296	0.046	0.178	0.150	0.387	0.296	0.048	0.098	0.204	0.155
S.E.	0.043	0.052	0.013	0.044	0.016	0.058	0.052	0.014	0.046	0.050	0.010
Standardised coefficient	0.089	0.083	0.075	0.082	0.175	0.086	0.083	0.075	0.043	0.076	0.304
p-value	<0.001	<0.001	0.001	<0.001	<0.001	<0.001	<0.001	0.001	0.035	<0.001	<0.001
R-square	0.035	0.041			0.139						

## Discussion

In a national representative sample of men and women aged 50 years and more our results show that a disadvantaged social position in childhood, measured as a father’s manual occupation, is associated with an increased risk of type 2 diabetes in adulthood. It has been shown also that a disadvantaged social position in childhood increases the odds of diabetes both directly and indirectly through an inflammatory score, adulthood social position and an adulthood risk score constructed from waist circumference, tobacco smoking and physical activity.

These findings need to be discussed further in the light of the literature reviewed earlier, but first several methodological issues must be addressed. Loss to follow-up of individuals between the waves of ELSA data collection might have introduced selection bias. Recent articles using ELSA data suggest that sample attrition is greater among those who were in a disadvantaged socioeconomic position at the start of the study. Demakakos et al. [14], meaning that our results are likely to underestimate the size of the real association between childhood social position and type 2 diabetes ( the comparison of the whole ELSA sample and our analytical sample suggests that any such underestimation may be small). In addition to attrition, the ELSA sample excludes those residing in institutions such as nursing homes. Although the proportion of such individuals in the population is small, we cannot claim that the ELSA sample is entirely nationally representative. On the other hand, ELSA’s large sample size is a major strength of the present study. Data imputation further maximised the effective sample size, and as such is major advantage of this paper in comparison to a previously published ELSA analysis on a similar topic [14]. Next, the diabetes variable was constructed mainly from self-reported information. Participants were asked to self-report whether they had been told by a doctor that they had diabetes mellitus or high blood sugar, whether they use pills or other medicines because of diabetes or high blood sugar, and whether they used insulin injections. Reliance on self-reports may have introduced reporting bias. Recall bias is also an issue in this analysis. As ELSA is a study of middle aged and older people, questions relating to childhood were asked several decades after the event, although any recall bias hopefully was minimised by the use of a computerised life-grid to collect the retrospective information. Further, residual confounding might be an issue. Although we have controlled for a range of relevant variables, there are still some variables, particularly data on diet, which would further improve the analytical model. The major methodological strength of this analysis, however, is the use of a pathway analytical approach to assess the links between childhood social position and type 2 diabetes in later life.

A recent paper reported, the association between socioeconomic characteristics in early life and diabetes mellitus in the English Longitudinal Study of Ageing using standard regression modelling [14]. Standard regression modelling techniques cannot precisely assess the mediating role of variables assumed to be on the pathway between exposures and outcomes; nor can they explicitly distinguish between the direct and indirect effects of such exposures. Our approach to the analysis, allowing evaluation of potential pathways, gives more insight into the relationship between childhood social circumstances and later diabetes. Finally, intermediary pathways between current social position and current risk factor score and inflammatory markers score could be correlated and, when tested, the effects of some pathways, such as via the inflammatory markers score, could be moderated and could become non-significant.

Despite some methodological limitations this analysis brings important findings. The estimated effect of childhood SEP is stronger than in most previous studies. It is possible that the use of data imputation and pathway analysis produced more precise measures, with smaller selection and recall biases, than previous studies which did not use such methods. For the population in the age range of interest to this analysis, this approach might be the best available in terms of data availability (the birth cohorts studies, with their prospective data from childhood, at present are younger than the ELSA respondents).

Our findings confirm the relationship between childhood socioeconomic position and adult type 2 diabetes mellitus and previously hypothesised pathways through current risk factors, current socioeconomic position and levels of inflammatory markers. The association between various measures of childhood socioeconomic position (education; income) and diabetes has been reported in some previous longitudinal studies of women [33,34], although not all [35]; and among men, such associations are reported as either weak or non-existent [10,33,35-37]. The previous analysis of ELSA similarly reported a weak association between childhood socioeconomic position and incident diabetes in women but no association in men [14]. However, their study focused on incident cases of diabetes within the study duration, and it is possible that diabetes cases in late life may be less strongly related to early childhood social circumstances than all cases including those at younger ages used in our analysis. Additionally, the number of incident cases of diabetes is limited in the ELSA dataset making estimates of the association between childhood socioeconomic position and incident diabetes relatively imprecise, while our analysis by using all cases and data imputation allowed more precise estimates.

Future replication of our findings in different cohorts is needed using a broader range of variables and focusing on other alternative pathways. The evidence from this study showing that childhood social circumstances influence health status in older age both directly and indirectly could provide strong arguments for policy makers to focus on socio-economic conditions of families with children aiming to reduce health inequalities across the entire life course.

## Conclusions

In conclusion, a disadvantaged social position in childhood is associated with an increased risk of diabetes in people aged 50 years and over, with social position in adulthood, health behaviours and inflammatory markers mediating this relationship. The direct role of childhood social conditions is reduced after taking account of these mediators, but it does not disappear entirely. Diabetes prevention programmes thus should concentrate on social disadvantage from early stages of life in addition to the established risk factors.

## Abbreviations

BMI: Body mass index; HPA: Hypothalamic-pituitary-adrenal; ELSA: English Longitudinal Study of Ageing; HbA1c: Glycated haemoglobin; SEP: Socioeconomic position; ESDS: Economic and Social Data Service; CI: Confidence interval.

## Competing interests

Authors declare there is no conflict of interest associated with this manuscript.

## Authors’ contributions

All three authors contributed substantially to the conception and design of the present paper. JP and GN obtained the data for the analysis, and analysed the data. JP drafted the initial manuscript. GN and DB critically revised the initial manuscript and all three authors participated in its further revisions. All authors read and approved the final version to be published. All authors agreed to be accountable for all aspects of the work.

## Pre-publication history

The pre-publication history for this paper can be accessed here:

http://www.biomedcentral.com/1471-2458/14/505/prepub
